# Editorial: NMR-based metabolomics

**DOI:** 10.3389/fmolb.2023.1337566

**Published:** 2023-12-18

**Authors:** Christophe Junot, Farhana R. Pinu, Justin J. J. van der Hooft, Sofia Moco

**Affiliations:** ^1^ Université Paris-Saclay, CEA, INRAE, Département Médicaments et Technologies pour La Santé (MTS), Gif-sur-Yvette, France; ^2^ Biological Chemistry and Bioactives Group, The New Zealand Institute for Plant and Food Research Ltd., Auckland, New Zealand; ^3^ Bioinformatics Group, Wageningen University & Research, Wageningen, Netherlands; ^4^ Molecular and Computational Toxicology, Chemistry and Pharmaceutical Sciences Department, AIMMS, Vrije Universiteit Amsterdam, Amsterdam, Netherlands

**Keywords:** metabolomics, NMR, metabolite, metabolism, sample preparation

Nuclear magnetic resonance (NMR) spectroscopy has a longstanding history in the analysis of biological small molecules (Jardetzky and Jardetzky, 1960). NMR is probably the most recognized spectroscopy able to provide decisive chemical information to elucidate molecular structures. With the rise of metabolomics, NMR entered a new area (Nicholson et al., 1999). The opportunity to use NMR to detect many metabolites in complex biological samples has led to important technology- and application-driven developments.

Improvements in sensitivity with the introduction of cryogenic probes, high magnetic field strengths, and automation during NMR acquisition have contributed to the robustness of modern high-resolution instruments. These hardware improvements were matched with many developments at the application level, e.g., dedicated pulse sequences allowing for flat baselines and efficient water suppression, and these have helped in the standardization of NMR procedures in metabolomics. Computational tools, including the availability of NMR spectral databases, have tremendously improved the consistency in metabolite identification and integration. As a result, “*NMR-based metabolomics*” is nowadays applied in a wide variety of fields from natural product research (Alfattani et al.) to clinical (Madrid-Gambin et al.) and biomedical applications (Zhong et al.).

NMR has managed to establish its niche within metabolomics as a result of important advantages, such as being robust, unbiased, and quantitative, remaining the spectroscopy to rely on for structure elucidation (Moco, 2022; Wishart et al., 2022).

Given the robustness and quantitative nature of NMR, the analysis of large human cohorts, including biobanks, has been an area of great expansion in “*NMR-based metabolomics*.” Given the sheer number of analyses, that can sometimes number in the 1,000s and often involve multiple centra of sampling and NMR acquisition, reproducibility and stability become central. The effect of various sample preparation protocols on the NMR quantification of 43 human plasma metabolites was assessed (Madrid-Gambin et al.). Four different approaches were tested: simple buffer addition was compared to ultrafiltration, protein precipitation with methanol, and glycerophospholipid solid-phase extraction (g-SPE) before buffer addition. While some of these methods seem more effective for protein and lipid removal from NMR spectra, differences in absolute concentrations were found. Hence, the complete and accurate reporting of procedures becomes of utmost importance when attempting to compare studies at the quantitative level.

While NMR may never compete with the sensitivity of mass spectrometry (MS), in combination, these two techniques are powerful in describing metabolomes (Alfattani et al.; Ghosh et al.; Zhong et al.). Combined techniques such as liquid chromatography (LC)-solid phase extraction (SPE)-NMR/MS have been around for a while; the combination with cryogenic electron microscopy (cryoEM) was discussed by Ghosh et al. as a strategy to further expand metabolite identification (Ghosh et al.). Microcrystal electron diffraction or microED is a cryoEM method that can precisely determine the structure of metabolites. From a complex natural product extract, a workflow integrating separation by LC, MS analysis, and SPE isolation of candidate metabolites, followed by parallel NMR acquisition and production of crystals for cryo-EM analysis is discussed.

Another strategy to enhance the capacity of metabolite identification is proposed by Alfattani et al. Semi-preparative LC time-slice fractionation before NMR acquisition allowed for the assembly of a “pseudo-LC-NMR” 2D contour map that could be paired with parallel LC-MS analyses. This high-resolution NMR-MS combination worked through molecular networks and unveiled novel metabolites present in the Mediterranean seagrass *Fusarium petroliphilum*. This approach will be useful in the identification of natural products in bioactive extracts of similar polarity (Alfattani et al.).

Imaging techniques are important to locate specific metabolic niches in biological samples associated with disease. Magic angle spinning (MAS)-NMR has been compared and combined with matrix-assisted laser desorption ionization (MALDI)-MS to image human cancers (Zhong et al.). The possibility of integrating both NMR and MS spectral data from human tissue images will assist their use in the diagnosis and classification of cancer types.

In summary, a series of original publications were gathered in this Research Topic, showcasing the multifaceted character of NMR. Various fields of research are presented, as well as the powerful alliance of NMR with MS in various applications toward improving metabolite identification, annotation, and structure elucidation. Taken together, “*NMR-based metabolomics*” is here to stay.
